# Increased intestinal permeability in primary Sjögren’s syndrome and multiple sclerosis

**DOI:** 10.1016/j.jtauto.2021.100082

**Published:** 2021-01-06

**Authors:** Bitte Sjöström, Anders Bredberg, Thomas Mandl, Lucía Alonso-Magdalena, Bodil Ohlsson, Shahram Lavasani, Mehrnaz Nouri, Gunnel Henriksson

**Affiliations:** a Department of Laboratory Medicine, Medical Microbiology, Lund University, Lund, Sweden; b Department of Rheumatology, Skåne University Hospital, Malmö, Lund University, Lund, Sweden; c Department of Clinical Sciences, Malmö, Rheumatology, Lund University, Malmö, Sweden; d Department of Neurology, Skåne University Hospital, Malmö/Lund, Lund University, Sweden; e Department of Clinical Sciences, Malmö, Neurology, Lund University, Sweden; f Department of Internal Medicine, Skåne University Hospital, Malmö, Sweden; g Department of Clinical Sciences, Malmö, Lund University, Sweden; h Department of Biology, Lund University, Lund, Sweden; i ImmuneBiotech AB, Medicon Village, Lund, Sweden; j Department of Clinical Sciences, Clinical Research Centre, Surgery Research Unit, Lund University, Malmö, Sweden; k Department of Clinical Microbiology, Skåne University Hospital, Lund, Sweden

**Keywords:** Primary Sjögren’s syndrome, Multiple sclerosis, Intestinal permeability, Faecal calprotectin, Lactulose/mannitol ratio

## Abstract

There is increasing evidence suggesting a role of intestinal dysfunction in a number of autoimmune diseases. Primary Sjögren’s syndrome (pSS) is a systemic autoimmune disease with a documented increased level of intestinal inflammation, whereas multiple sclerosis (MS) is an organ-specific autoimmune disease known to exhibit increased intestinal permeability. In this study we determine to what extent intestinal inflammation, analysed by a faecal calprotectin ELISA, is accompanied by altered intestinal wall permeability, as measured by a lactulose and mannitol intestinal absorption assay. Intestinal permeability was increased in both pSS and MS patients, while faecal calprotectin was elevated in pSS but normal in MS. Our findings suggest different mechanisms mediating a leaky gut in these two diseases: in pSS there is autoimmune attack directly on the intestinal wall; in MS, with autoimmunity being limited to the CNS, it may be due to a disturbed CNS regulation of enteric nerve function.

## Highlights

• Intestinal permeability analysed by sugar absorption is increased in primary Sjögren’s syndrome and multiple sclerosis.
• Intestinal wall inflammation as determined by faecal calprotectin is observed in pSS but not in MS.
• Leaky gut may be due to autoimmune targeting of the intestinal wall in pSS and to disturbed intestinal innervation in MS.

## Credit author statement

Bitte Sjöström, Anders Bredberg, Thomas Mandl, Lucia Alonso-Magdalena, Shahram Lavasani and Gunnel Henriksson took part in conception and design of the study. Bitte Sjöström, Shahram Lavasani, Mehrnaz Nouri and Gunnel Henriksson acquired the results. Bitte Sjöström and Gunnel Henriksson had access to all data and take responsibility for the accuracy of the data analysis. Bitte Sjöström, Anders Bredberg, Thomas Mandl, Lucía Alonso-Magdalena, Bodil Ohlsson, and Gunnel Henriksson were involved in drafting the article.

## 1 Introduction

In recent years, there has been a veritable explosion of reports showing a correlation between intestinal microbiome alteration and a number of both organ-specific and systemic autoimmune disorders [[1], [2], [3], [4], [5], [6]]. Primary Sjögren’s syndrome (pSS) is a systemic autoimmune disease affecting exocrine glands and with symptoms from many organs, including the gastrointestinal tract [3]. Multiple sclerosis (MS) is also an autoimmune disease, but in which the organ-specific, immune-mediated disease process results in demyelination and neurodegeneration of the central nervous system (CNS) [7]. Both MS and pSS have in previous studies been shown to have an altered intestinal microbiome [3,4]. MS has also been reported to have an increased intestinal permeability (IP) [8], whilst in pSS no data on IP have been published.

The ratio in urine between orally taken lactulose and mannitol (L/M) is a non-invasive measure of IP [9]. The protein zonulin plays an important role in the disassembly of the tight junctions, which link the intestinal epithelial cells to each other, and has been considered to be a marker of IP, however, this has later been put into question [10]. Faecal calprotectin (FC) is an inflammatory protein emanating from leukocyte shedding into the intestinal lumen, and its concentration is routinely used in the clinic as a marker of inflammatory bowel disease activity [11].

In this study we wanted to determine if the reported intestinal inflammation in pSS [12] is accompanied by altered intestinal wall permeability. MS, shown to have an increased IP, was chosen as a control autoimmunity disease with potential to provide information on the mechanism of intestinal derangement in pSS.

## 2 Materials and methods

### 2.1 Study design

This is a prospective study on consecutive outpatients, primarily with scheduled visits, at our university hospital’s Departments of Rheumatology and Neurology. Patients previously diagnosed with pSS (n ​= ​20) or MS (n ​= ​18) were recruited. The pSS diagnosis was made according to the American–European Consensus Group criteria [13] and the relapsing-remitting MS patients were diagnosed by using McDonald criteria [14]. The 10 healthy controls (HC) were recruited amongst friends and university staff. Characteristics of the patients and HC are described in Table 1, showing that all MS subjects were treated with natalizumab. This study was performed according to the Declaration of Helsinki and approved by the Regional Ethics Committee, Lund, Sweden (reference number 2015/29). All subjects gave written informed consent before inclusion in the study.

**Table 1:** Characteristics of study participants.

	pSS (n ​= ​20)	MS (n ​= ​18)	HC (n ​= ​10)
Age, years	62 (40; 68)	42 (39; 50)	45 (27; 62)
Males/females	3/17	4/14	4/6
Disease duration, years	8 (2; 16)	9 (2; 14)	
Treatment at time of study:			
Glucocorticoids	2	0	0
Anti-malarials	4	0	0
Glucocorticoids ​+ ​anti-malarials	2	0	0
Glucocorticoids ​+ ​rituximab	1	0	0
Anti-malarials ​+ ​rituximab	1	0	0
Natalizumab	0	18	0

HC, healthy controls; MS, multiple sclerosis; pSS, primary Sjögren’s syndrome.

Values are given as median (interquartile range), number or percentage of subjects.

### 2.2 Lactulose/mannitol ratio analysis

Urine and a spoonful of stool was collected from each study subject, and kept at −20 ​°C until analysis. For the IP assay, the participants were asked not to take non-steroidal anti-inflammatory drugs (NSAID) for at least one week prior to testing and to refrain from alcohol for at least 3 days. After 8 ​h fasting (overnight), the participants urinated, then drank a 200 ​mL solution containing lactulose (10 ​g) and mannitol (5 ​g), followed by 300 ​mL of water. The volume of urine collected over the next 6 ​h was recorded, and 10 ​mL was transferred to a tube and stored until analysis. Urine lactulose and mannitol concentrations were measured by the EnzyChromTM Intestinal Permeability Assay Kit (EIPM-100), (BioAssay Systems, Hayward, USA). We chose 0.018 of L/M as the upper limit of normal, since all our controls were at or below this level.

### 2.3 Calprotectin analysis

FC concentration was measured by using an enzyme-linked immunosorbent assay (ELISA) kit (Immundiagnostik AG, Bensheim, Germany). An FC level higher than 100 ​μg/g is used in clinical practice as a marker of disease activity in inflammatory bowel disease patients [15] and we consider such a result to indicate strong intestinal inflammation. All ELISA testing was carried out with duplicate wells and blinded.

### 2.4 Zonulin analysis

Zonulin concentration in faeces was determined with an ELISA kit (Immundiagnostik AG), as described by the manufacturer with addition of 0.75 ​mL buffer per 15 ​mg faeces.

### 2.5 Statistics

Median and interquartile range (IQR) were used for descriptive data. For comparison of levels of clinical markers between groups, the Mann–Whitney U test was used. Spearman’s correlation coefficient was used for the scatterplot correlation analysis. *P* values ​< ​0.05 were considered statistically significant.

## 3 Results

**Fig. 1:**
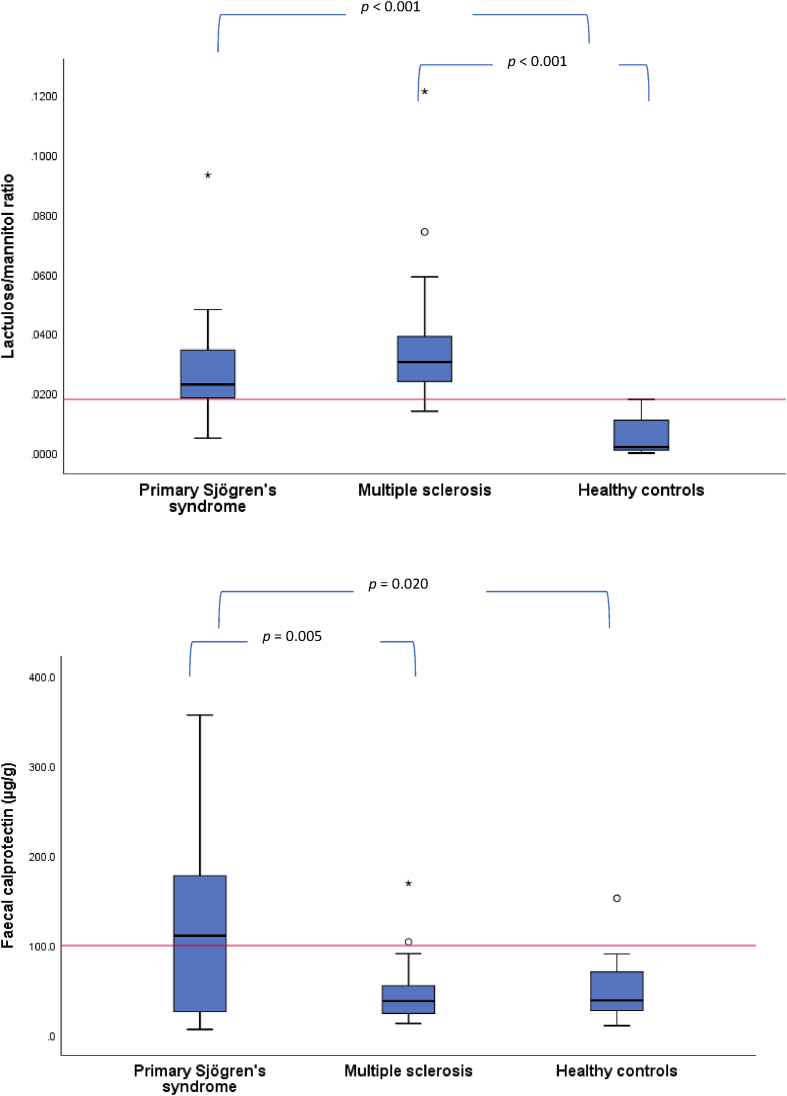
(A) Lactulose/mannitol urine concentrations ratio serving as a measure of intestinal permeability. The red line indicates the cut off. (B) Faecal calprotectin serving as a measure of intestinal inflammation. The red line indicates the level of 100 ​μg/g clinically used as a marker of intestinal inflammation in inflammatory bowel disease. Boxes represent values between quartiles 1 and 3, and a thick line indicates the median. Whiskers show the max and min values located above the top or below the bottom of the box, respectively, within a 1.5 interquartile distance. Circles denote outliers values located outside a 1.5 interquartile distance, and stars denote outliers located outside a 3 interquartile distance. Two extreme primary Sjögren’s syndrome outliers at 1113 and 2037 ​μg/g are not shown, but are included in the calculation of the box and whiskers. (For interpretation of the references to colour in this figure legend, the reader is referred to the Web version of this article.)

IP was measured as the ratio between lactulose and mannitol in urine, after oral ingestion of these two non-metabolized sugars. Lactulose is a large and normally non-absorbable molecule passively diffusing through a disturbed intestinal barrier, while the smaller mannitol is actively absorbed. The L/M ratio was significantly increased, indicating an increased intestinal permeability, in both the patient groups as compared with HC (pSS *p* ​< ​0.001 and MS *p* ​< ​0.001) but there was no difference between pSS and MS (Fig. 1A and Table 2). 14 of the 19 analysed pSS patients (for one patient L/M could not be determined due to loss of the urine during storage), and 15 of the 18 with MS had a ratio result exceeding our 0.018 cut-off value.

**Table 2:** Results for intestinal permeability measured by a lactulose and mannitol intestinal absorption assay, and for faecal calprotectin and zonulin analysis.

	pSS (n ​= ​20)^a^	MS (n ​= ​18)	HC (n ​= ​10)	*p* values
				pSS-MS: >0.05
**Lactulose/mannitol ratio**	0.023 (0.018; 0.035)	0.031 (0.024; 0.040)	0.002 (0.001; 0.012)	pSS-HC: <0.001
				MS-HC: <0.001
				pSS-MS*:* 0.005
**Faecal calprotectin (μg/g)**	111 (25.5; 195)	38.1 (24.3; 61.8)	38.9 (24.1; 75.7)	pSS-HC: 0.020
				MS-HC: >0.05
				pSS-MS: >0.05
**Faecal zonulin (ng/mL)**	103 (73.5; 143)	134 (82.5; 194)	105 (81.6; 132)	pSS-HC: >0.05
				MS-HC: >0.05

HC, healthy controls; MS, multiple sclerosis; pSS, primary Sjögren’s syndrome.

Values are given as median (interquartile range).

a For one pSS patient lactulose/mannitol ratio could not be determined due to loss of the urine during storage.

The FC concentration was significantly elevated only in the pSS patients, as compared to MS (*p* ​= ​0.005) (with all MS subjects being treated with natalizumab) and HC (*p* ​= ​0.020) (Fig. 1B and Table 2). Two extreme pSS outliers with FC values of 1113 and 2037 ​μg/g are not shown but were included in the statistical calculation.

The analysis of faecal zonulin showed similar medians for the three study groups, with no significant difference (Table 2).

**Fig. 2:**
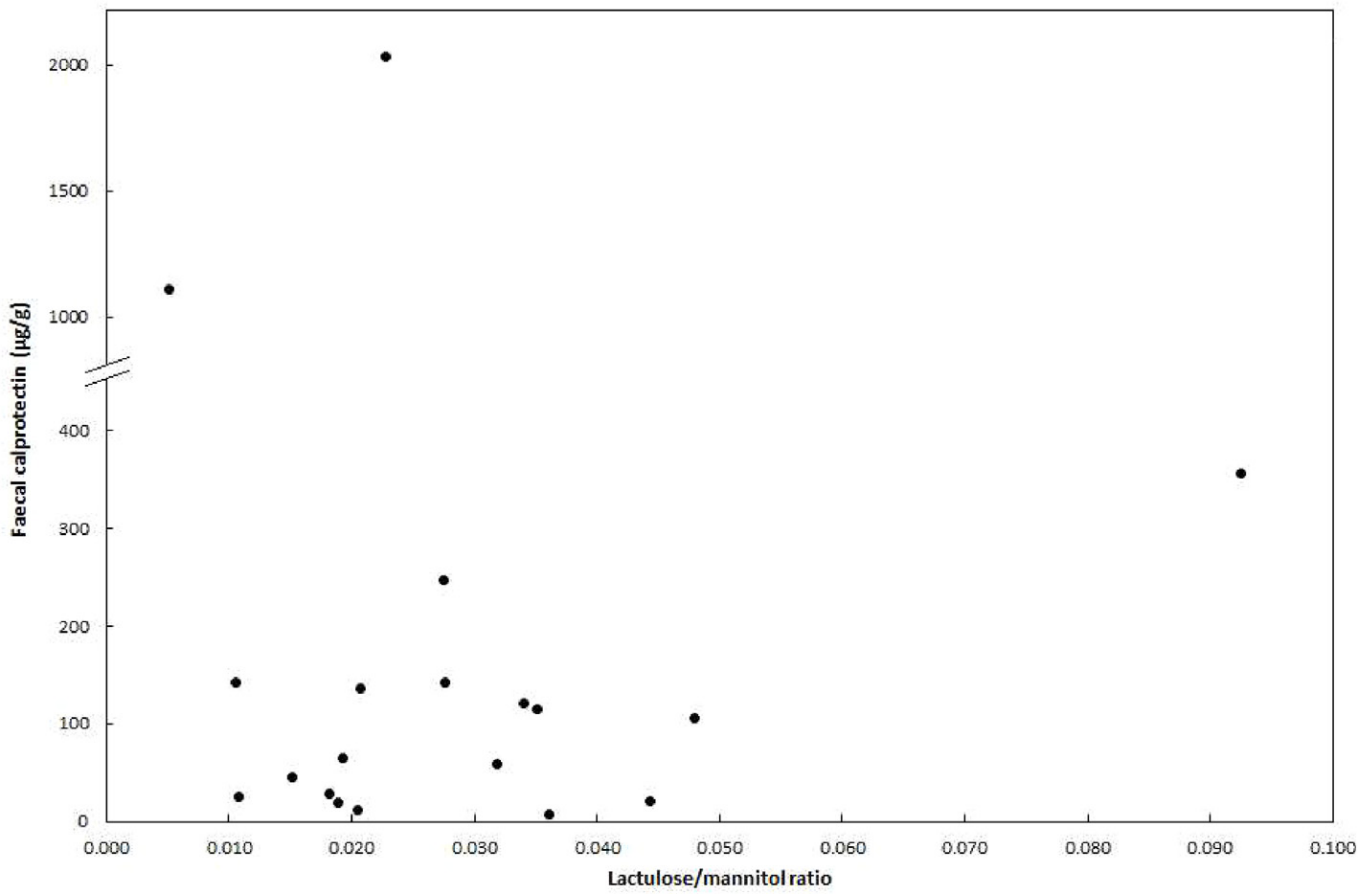
Scatterplot with the results on lactulose/mannitol ratio and faecal calprotectin displayed for each individual primary Sjögren’s syndrome patient. Note that the Y-axis is split at 400 ​μg/g faecal calprotectin, in order for two extreme outliers to be displayed.

We found no correlation between any pair of our three biomarkers within any of the subject groups, including L/M ratio and FC which were both clearly elevated in pSS; a Spearman regression analysis showed r ​= ​0.008 and *p* ​= ​0.974. This lack of significant correlation is evident also from the scatterplot, where the points form a swarm pattern rather than a straight line (Fig. 2). The scatter plot shows that amongst the 14 pSS patients with an increased L/M ratio, there were eight with a high FC level indicating intestinal inflammation, while the remaining six had a low FC value (<65 ​μg/g) (Fig. 2). Similarly, the rare MS and HC cases with FC elevation displayed no apparent correlation with L/M; in MS one of the two patients with increased FC had an elevated L/M, and the single HC subject had a normal L/M ratio as low as 0.002 (MS and HC results are not shown as a scatterplot). A high FC level was present in two of the five pSS individuals with no L/M abnormality. These two patients had a history of autoimmune hepatitis (FC level: 142 ​μg/g) and gastric immunocytoma (FC level: 1113 ​μg/g), respectively, suggesting that a diagnosis separate from pSS may have influenced the FC level.

## 4 Discussion

We report two new findings: increased IP in pSS and a normal level of FC in MS (notably with all MS subjects being treated with natalizumab, being discussed below). In addition, we confirm previous data by showing an elevated FC concentration in pSS [12] and increased IP in MS [8]. Thus, in pSS there is both inflammation and an impaired IP, whilst MS has the same level of increased IP as seen in pSS but without inflammation.

There was no difference in results between the 10 treated and the nine non-treated pSS patients on the result for IP (*p* ​= ​0.22) or FC (*p* ​= ​0.57). One limitation of our study is that all the MS patients were on treatment with natalizumab. The mechanism of action of natalizumab includes binding to an integrin cell adhesion molecule on the surface of T lymphocytes and inhibiting their passage across the blood brain barrier as well as their infiltration of the gut mucosa [16], which may have influenced our results. However, it has been reported that IP is increased in treatment-naive MS patients to a similar extent to that observed by us, so a potential role of natalizumab seems less likely [8]. Intestinal calprotectin originates from mucosal granulocytes and antigen-presenting cells, but not from T lymphocytes [17]. While we do acknowledge that it can be postulated that natalizumab is capable of healing an intestinal membrane damaged by autoreactive gut T lymphocytes, and secondarily lead to loss of activated granulocytes, we consider it unlikely that this therapy significantly has influenced our observations of a fully normal FC level coupled with a strongly elevated IP in MS. Two weaknesses in our work are the low number of study subjects and the limited matching of the three study subject groups for age and sex. However, although FC concentration is known to be age-dependent [18], it is unlikely that the higher age among pSS compared to both MS and HC, significantly influenced our results. HC under 60 years of age had an average of 38 ​μg/g compared to 192 in pSS, and at age 60+ the values were for HC 112 ​μg/g and pSS 298 ​μg/g; and there was no correlation between age and FC levels in pSS. We therefore conclude that our findings reflect the pSS and MS disorders *per se* rather than being related to sex or age.

To what extent can the present report shed any light on whether intestinal pathology in pSS and MS reflects cause of disease or appear merely as a consequence of it? One interpretation can be that the distinctly different results seen in pSS and MS suggest that a main gastrointestinal pathogenetic causative event in pSS is inflammation, whereas in MS it is a disturbed enteric nervous system (ENS). However, when viewed in a wider perspective there are numerous alternative explanations. Until only a few years ago, it was commonly thought that immune cross-reactivity between enteric bacteria and self-epitopes initiates many autoimmune diseases [19], but an understanding of the pathogenesis of autoimmunity has become vastly more challenging with evidence emerging documenting an interplay and cross-talk involving neurons, immune cells and the intestinal mucosa and microbiome [[1], [2], [3], [4], [5], [6]]. Examples of recent reports illustrating this complexity include inhibition of antigen-presenting cells and stimulation of intestinal exocrine glands by the release of the neurotransmitter acetylcholine from both the vagal nerve and CD4^+^ T cells [20]. Another example is propionic acid emanating from a limited set of species in the gut microbiome, reaching the CNS and providing clinical improvement in MS patients [21].

Relapses in MS are thought to be mainly mediated by aberrantly activated and/or insufficiently regulated pro-inflammatory CNS-specific effector T-cells, that traffic to the CNS parenchyma and cause injury [7]. However, even if the autoimmune activity is directed against CNS, gastrointestinal alterations have been reported, including a reduced repertoire of the gut microbiome (often referred to as dysbiosis) [4,5] and an increased IP [8]. It is known that it is part of normal physiology that the CNS exerts a major influence on the intestinal wall, by neural circuits controlling both the vagal nerve and, more directly to the gut, the ENS [6]. With this knowledge at hand, it is conceivable that there is no autoimmune target located within the intestinal wall, providing no potential for tissue damage, granulocyte and monocyte infiltration, and calprotectin production. Instead, an altered ENS may lead to mucosal reorganization and an altered IP. This idea is supported by a recent report showing that gut neuron activity causes the increased IP appearing during salmonella infection [22].

In contrast to MS, in pSS a systemic autoimmune activity is thought to be linked to the multitude of organs affected. The documented gastrointestinal tract effects include a dysbiosis pattern similar to that seen in MS [3] and increased levels of FC correlating with severity of intestinal symptoms [12]. It has been suggested that this gut pathology is mediated by destruction and/or reduced secretory function of intestinal exocrine glands and forming an analogy with the increased saliva calprotectin concentration in this pSS syndrome hallmarked by salivary gland pathology [23]. Because the autonomic nervous system is also affected in pSS [24], it may be argued that an autoimmune effect on the ENS can also contribute to the intestinal changes. As said for MS, the finding that the ENS can modulate IP supports this argument [22]. In further contrast with MS, tissue damage and inflammation with calprotectin production is an expected consequence.

In conclusion, our findings indicate that there is a leaky gut in both pSS and MS patients, and the FC elevation in the pSS group suggests that the mechanism behind the IP disturbance involves intestinal wall inflammation in pSS whilst not in MS. We discuss to what extent our findings support the idea that in pSS there is autoimmune attack directly on the intestinal wall, whilst in MS, with autoimmunity being limited to the 10.13039/100000144CNS, the disturbance of intestinal wall function is secondary to an alteration of ENS which may be CNS-mediated.

## Author contributions

BS, AB, TM, LAM, SL and GH took part in conception and design of the study. BS, SL, MN and GH acquired the results. BS and GH had access to all data and take responsibility for the accuracy of the data analysis. BS, AB, TM, BO, LAM and GH were involved in drafting the article. All authors revised the manuscript critically and approved the final version for submission.

## Funding

This study was supported by research grants from the 10.13039/501100007949Swedish Rheumatism Association, the Swedish Society of Medicine, and Region Skåne’s R&D funds. The funders had no involvement in any aspect of the study and writing of the report.

## Declaration of competing interest

The authors declare that they have no known competing financial interests or personal relationships that could have appeared to influence the work reported in this paper.
